# Determination of unique power conversion efficiency of solar cell showing hysteresis in the I-V curve under various light intensities

**DOI:** 10.1038/s41598-017-10953-3

**Published:** 2017-09-18

**Authors:** Ludmila Cojocaru, Satoshi Uchida, Koichi Tamaki, Piyankarage V. V. Jayaweera, Shoji Kaneko, Jotaro Nakazaki, Takaya Kubo, Hiroshi Segawa

**Affiliations:** 1 Research Center for Advanced Science and Technology, The University of Tokyo, Komaba 4-6-1, Meguro-ku, Tokyo, 153-8904 Japan; 2 Komaba Organization for Educational Excellence, Faculty of Arts and Sciences, The University of Tokyo, Komaba 3-8-1, Meguro-ku, Tokyo, 153-8902 Japan; 3 SPD Laboratory, Inc., Johoku 2-35-1, Naka-ku, Hamamatsu, 432-8011 Japan; 4 Department of General Systems Studies, Graduate School of Arts and Sciences, The University of Tokyo, Komaba 3-8-1, Meguro-ku, Tokyo, 153-8902 Japan

## Abstract

Energy harvesting at low light intensities has recently attracted a great deal of attention of perovskite solar cells (PSCs) which are regarded as promising candidate for indoor application. Anomalous hysteresis of the PSCs a complex issue for reliable evaluation of the cell performance. In order to address these challenges, we constructed two new evaluation methods to determinate the power conversion efficiencies (*PCEs*) of PSCs. The first setup is a solar simulator based on light emitting diodes (LEDs) allowing evaluation of the solar cells at wider range of light intensities, ranging from 10^2^ to 10^−3^ mW·cm^−2^. As the overestimate error, we found that the *PCEs* of dye sensitized solar cell (DSC) and PSCs increase dramatically at low light intensities conditions. Due to the internal capacitance at the interfaces on hybrid solar cells, the measurement of current below 10^−2^ mW·cm^−2^ shows constant value given high *PCE*, which is related to the capacitive current and origin of the hysteresis. The second setup is a photovoltaic power analyzing system, designed for tracking the maximum power (*﻿P*_max_) with time. The paper suggests the combination of the LED solar simulator and *P*_max_ tracking technique as a standard to evaluate the *PCE* of capacitive solar cells.

## Introduction

Efficient power generation under weak irradiation is essential for indoor applications or installation and installation in cloudy places. However, solar cells performances is usually evaluated by solar simulators with 10^2^ mW·cm^−2^ irradiance (AM1.5 G) as described in IEC 60904-3 etc., as seen in the solar cell efficiency tables^1^. This condition (1 sun) is almost equivalent to direct sunlight at AM1.5 G. In the case of dye-sensitized solar cells (DSCs) and perovskite solar cells (PSCs), better performances have been reported under weak irradiation conditions^2–6^. Performance measurement of solar cells at very low irradiance levels is not well established yet, their measurement conditions depended largely on the measurement parameters. Establishing a standardized method for evaluation under weak irradiation is a necessary step for reliable reports on *PCE* of solar cells designed for indoor application.

The general method to determine the *PCE* of solar cells is to measure the photocurrent by scanning the bias potential. The results are expressed as a current density – voltage (*J*-*V*) curve or an output electric power – voltage (*P*-*V*) curve, and the ratio of the maximum output power (*P*_max_) to the irradiation intensity is described as the conversion efficiency (*η*). Therefore, accurate current – voltage (*I*-*V*) measurement is essential for the evaluation of solar cells. However, the anomalous *I-V* hysteresis of PSCs^7–9^ is a problem for reliable evaluation of the *PCE*. Since different *I*-*V* curves are obtained depending on the voltage scan direction, two efficiency values are obtained for a device. The recent rapid rise of PSCs, which exhibit *PCEs* more than 22%^10^, demands accurate determination of the *PCE* values. There are reports on evaluation the *PCE* by continuous measurement of photocurrent under applied bias potential, where *P*_max_ was obtained^11,12^. However, the temporal change in *P*_max_ could not be followed. A maximum power point tracking (MPPT) technique would be a promising approach to track such changes. MPPT is a well-established technique for maximizing power output of large solar arrays. Adequate adjustments are necessary for the application of MPPT to precise measurements of small size solar cells including also hysteretic devices. Before the measurement, careful calibration is carried out by using aluminum box unit to cancel the road and impedance of electric wire and contact resistance under the shield from electromagnetic waves.

In this study, two machines have been designed and constructed to determine the unique power conversion efficiency of solar cells showing hysteresis during *I-V* measurements under various light intensities.

The first machine consist is a solar simulator using light emitting diode (LED) as illumination source instead of the conventional Xe lamps. In generally, Xe lamps are used as light source in almost all solar simulators because of its reasonably good match with the solar spectrum. However, large power consumption, illumination instability with aging and relatively short lifetime are shortcomings of using Xe lamps as light source. In contrast, LED has several advantages over the present Xe lamps, such as long operating life and high-energy efficiency, and has therefore been previously proposed as an alternative light sources for solar simulators^13^. The use of a LED-based solar simulator allows evaluation at wider range of light intensity from 10^2^  to 10^−3^ mW·cm^−2^. The light intensity is further decreased (from 10^−3^ to 10^−4^ mW·cm^−2^) by combining three different stainless steel meshes as fading filters.

The second machine is a photovoltaic power analyzing system based on the MPPT technique to provide trustworthy evaluation of *PCE* for solar cells and in particular PSCs under varying light intensities controlled by the LED solar simulator.

## Results and Discussion

### Evaluation of the solar cells using LED solar simulator

In order to confirm the adjustment of the light intensity, we have measured *I-V* curves for c-Si solar cell as a reference, PSC and DSC at different light intensity conditions, ranging from 10^2^ to 10^−4^ mW·cm^−2^.

**Figure 1:**
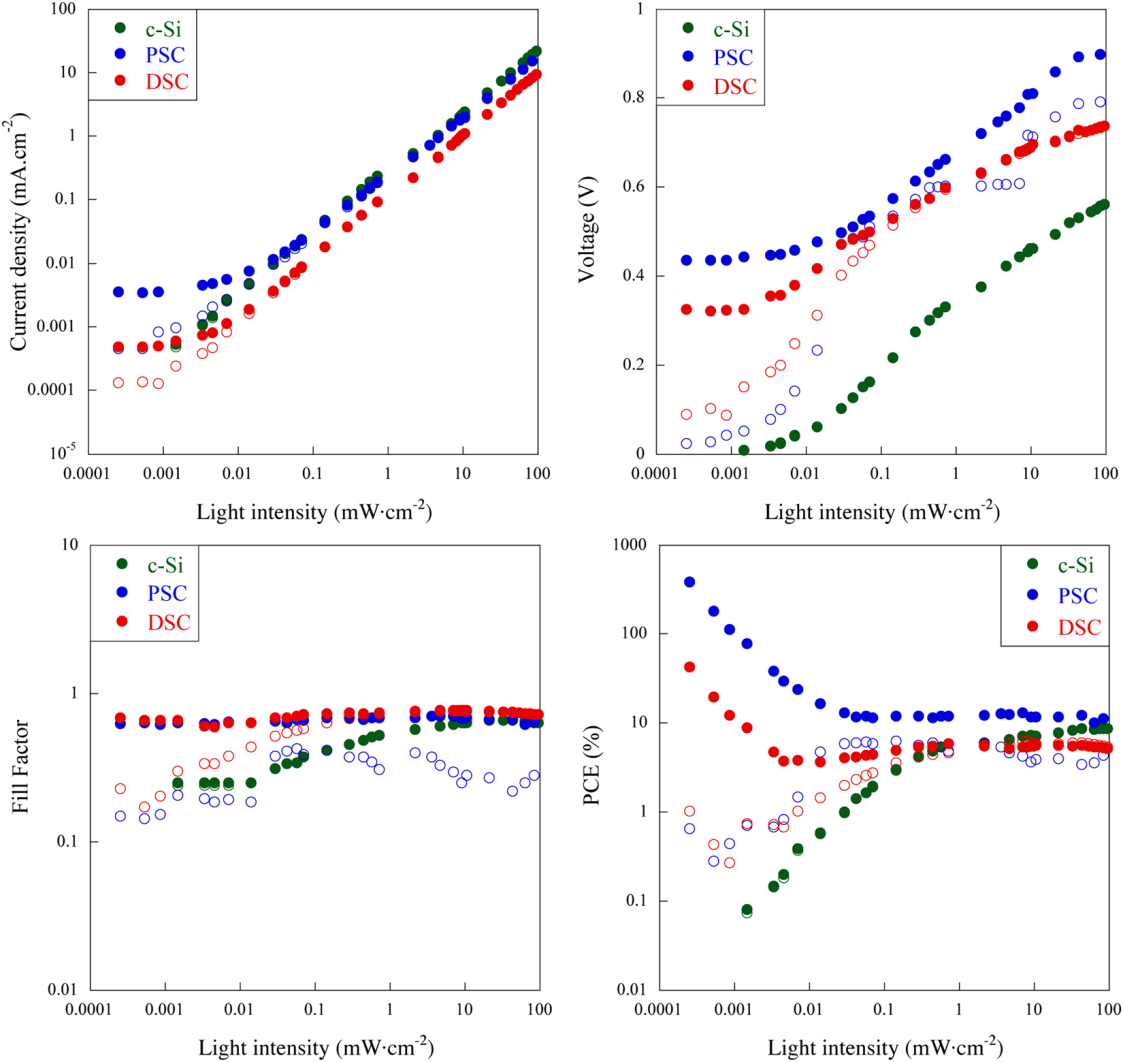
Evaluation of the photovoltaic parameters of the c-Si solar cell (green curves), DSC (red curves) and PSC (blue curves) at different light intensity, closed circles: reverse scan condition and open circles: forward scan condition.

The photovoltaic parameters for both the reverse and forward scan conditions (short-circuit density (*J*_*sc*_), open-circuit voltage (*V*_*oc*_), fill-factor (*FF*), power conversion efficiency (*PCE*)) were extracted from the *I-V* curves and have been plotted in Fig. 1. The *I-V* curves of PSC and DSC shows correct shape and reproducible during the evaluation (Supplementary Fig. 1).

The variation in illumination intensity significantly affects the *J*_*sc*_. Changes in the illumination intensity between 10^2^  to 10^−2^ mW·cm^−2^ causes a proportional change in the *J*_*sc*_ for all solar cells. The trend observed in *J*_*sc*_ is directly proportional to the number of the photons absorbed by the semiconducting material, as we can see from Fig. 1. Similar results has been reported in numerous works, but in a more limited light intensity range^14–16^. At the light intensities below 10^−3^ mW·cm^−2^, the current from the c-Si solar cell cannot be measured due to lack of any photoresponse of the device. However, DSC and PSC solar cells show constant non-zero value of the *J*_*sc*_ even below 10^−2^ mW·cm^−2^. The constant *J*_*sc*_ is unlikely to be from a irradiation at such low intensities because the value remains constant for all intensities below 10^−2^ mW·cm^−2^. We believe this current originates from a discharging internal capacitor formed at one of the interfaces in the multilayered structure of PSC and DSC.

### Capacitive current observed at low light intensity conditions

Solar cell that shows internal capacitor effect has two different paths for reveres and forward *I-V* scans. The hysteresis and the effect of the capacitive behavior on the performance of solar cells is well known and already reported^17–20^. Several variation of the dynamic models exist, for example one or two diode models including one or two capacitances (Supplementary Fig. 2) for electronic simulation of the *I-V* hysteresis is used^19,21,22^.

The scheme shown in the Supplementary Fig. 3 visualize the current flow and effect of capacitor charging and discharging during the *I-V* measurement. The cell output voltage is changed to a set of discreet values by changing the effective load during the *I-V* scan. *V*_*set*_ is a variable that represents the fixed voltage values set by the *I-V* measuring instrument for each data point in the *I-V* curve. Current output (*I*_*output*_) represents the measured current values at each *V*_*set*_. *I-V* curves can be divided into three main sections depending on the quadrant of the Cartesian plane (Supplementary Fig. 3). When analyzing reverse and forward *I-V* curves, six different stages should be considered. A strong *I-V* hysteresis appears on the quadrant *I*, were *V*_*set*_ < *V*_*oc*_. When a capacitor is connected to the DC power supply the current flows through the circuit. Based on that, the current flows out from the cell and the internal capacitor is discharging in the reverse scan condition (*V*_*set*_ < *V*_*oc*_). In this case the *I*_*output*_ can be expressed by formula 1. In contrast, the internal is capacitor is charging in the forward scan condition and the *I*_*output*_ can be expressed by the formula 2. Following formula 1 and 2, the current output of the reverse scan shows higher values than that of forward scan conditions.

1 $${I}_{output}={I}_{photo}+{I}_{cap.disch\text{arg}e}$$

2 $${I}_{output}={I}_{photo}-{I}_{cap.ch\text{arg}e}$$

At reasonable high light intensity the photo generated current originates from the excitation of light absorber in solar cells, but at low light intensity the generated current from the capacitance adds to the total output current and gives rise to an erroneous value. That is the main reasons as to why the photocurrent at low light intensity is non-zero, and wrongly evaluated in DSC and PSC. Therefore, the *PCE* is wrongly evaluated especially in high capacitive solar cells.

Considering equations 1 and 2 the calculated total current output of the devices is expected to be close to the zero at low light intensity. However, this is not the case for DSC and PSC as can be seen from *I-V* curves. The current registered can be assigned as capacitive current originated as a consequence from a dynamic charge-discharge process taking place at the interfaces in the PSC and DSC. The behavior of the charge transfer process depends on the scan rate and direction of the *I-V* scan^20^.

### Capacitance calculation using low light intensity I-V data

To evaluate the capacitance of PSC and DSC we have used Formula 3. The flow of the electrons “through” a capacitor is directly proportional to the rate of voltage change across the capacitor. The relationship between the current “trough” the capacitor and rate of voltage change across the capacitor can be expressed as:

3 $$C=I({\rm{\Delta }}t/{\rm{\Delta }}V)=I/s$$

were the *I* is the current, *C* is the capacitance and *s (ΔV/Δt)* is the scan rate.

For the calculation of the capacitance the value of the capacitive current was extracted from the reverse scan condition (discharge current, *J*_*cap.discharge*_) seen in Fig. 1a at light intensity of 8.58 × 10^−4^ mW·cm^−2^. Calculated data are shown in the Table 1.

**Table 1:** Calculated capacitance for solar cells, current extracted from reverse scan condition at 8.58 × 10^−4^ mW·cm^−2^ light intensity.

Sample	*J*_*cap.discharge*_ (A·cm^−2^)	Scan rate(V·s^−1^)	Capacitance (μF·cm^−2^)
c-Si	0	0.1	0
PSC	3.79 × 10^−6^	0.1	37.9
DSC	4.66 × 10^−7^	0.1	4.66

The measurement indicates that the DSCs and PSCs have internal capacitor effects, for DSC we found that the capacitance equal is 4.66 μF·cm^−2^, which is one order of magnitude lower that the capacitance of PSC, 37.9 μF·cm^−2^. We believe that this difference originates from the less pronounced hysteresis observed for the DSC compared to more pronounced hysteresis observed for PSC. Moreover, the value of the experimental calculated capacitance for PSC is slightly smaller compared to the value, we have previously reported (250 μF·cm^−2^)^21^. This is also related to the difference of the *I-V* measurements conditions.

The capacitance calculated from the relation between the discharge current and scan rate for actual PSC at 6.98 × 10^−3^ mW·cm^−2^ (Supplementary Fig. 4) shows a well-known downward trend with increasing scan rate, this behavior can be explained by the charge-discharge process observed for supercapacitors^23,24^. In addition, the discharge capacitive current increase with increasing scan rate which affects the total output current (formula 1). At high scan speed conditions the total output current is higher that at low scan speed conditions. PSC solar cells can be categorized as high capacitive devices.

*V*_*oc*_ varies with illumination intensity and decreases more drastically for c-Si cell than the for DSC and PSC cases. At low light intensity conditions (below 10^−1^ mW·cm^−2^) PSC and DSC can maintain high photovoltage. An difference in *V*_*oc*_ for reverse and forward scan conditions can be observed only in the case of DSC and PSC at low light intensity. The *V*_*oc*_ extracted from the *I-V* curves shows wrongly evaluated due to the erroneous evaluation of the photocurrent at low light intensity below 10^−2^ mW·cm^−2^.

From this experiment, we have seen that the *PCE* of c-Si solar cell is strongly dependent on the light intensity conditions and shows a dramatically decrease of the efficiencies with decreasing light intensity from 10^2^ to 10^−2^ mW·cm^−2^. However, the *PCE* of DSC and PSC cells is less affected to the change in the light intensity for this range from 10^2^ to 10^−2^ mW·cm^−2^. Below 10^−2^ mW·cm^−2^, the power efficiencies improve to over 100% for perovskite and over 10% for dye cells. This is due to the charge/discharge current into the capacitive element in the devices not by real photo generated one. Several groups have already reported high power conversion efficiency for DSCs and PSCs for low light intensity condition^2–6^. In our previous report^21^, we have shown how that PSC cells can exhibit high capacitance at the interface of the CH_3_NH_3_PbI_3_ in contact with TiO_2_ and spiro-OMeTAD. The hysteresis observed in simulated *I-V* curves based on the equivalent circuit (Supplementary Fig. 2) containing two capacitances at the interfaces shows good matching with experimental *I-V* curves^21^.

### Evidence of the capacitance effects from the physical constructed device

**Figure 2:**
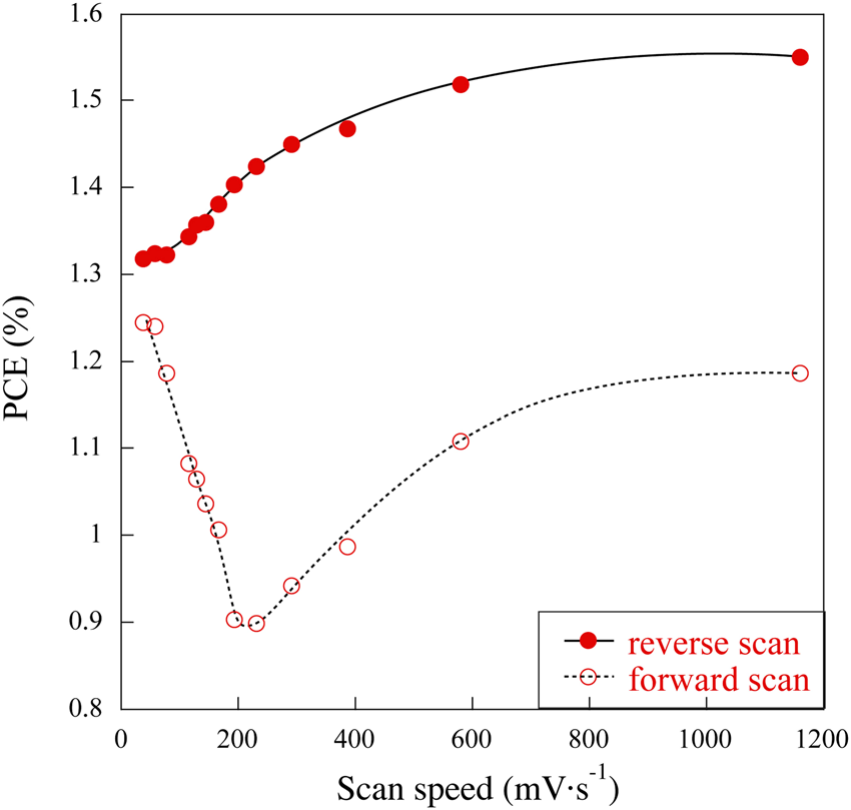
*PCE* for reverse and forward scan measurement extracted from the measured *I-V* curves at 10^2^ mW·cm^−2^ of the physical device.

Based on the equivalent circuit (Supplementary Fig. 2) we have constructed physical device composed of two silicon cells with two capacitances and resistor (Supplementary Fig. 5). In order to conform the capacitive current, low light intensity *I-V* measurement of the device were carried out. The dependence of the *J*_*sc*_ and *PCE* with light intensity for device based on two diodes and two capacitors are shown in the Supplementary Fig. 6. *J*_*sc*_ decrease with decreasing light intensity from 10^2^ to 10^−1^ mW·cm^−2^. A similar trend was observed below 10^−2^ mW·cm^−2^ (Fig. 1), the non-zero current observed at this light intensity is attributed to capacitive current. It has been found that the *PCE* of the device increase drastically at light intensities below 10^−2^ mW·cm^−2^. This phenomena provide additional evidence that the high capacitances values of the devices leads to erroneous evaluation of the photocurrent and the *PCE* at low light intensity conditions. In order to further investigate that, we measured the *I-V* curves at different scan speed conditions at 10^2^ mW·cm^−2^ illumination (Fig. 2) of this physical device.

As shown in the Fig. 2, the *PCEs* are affected by the scan speed rate used for *I-V* measurement. At faster scan speeds conditions the *PCE* shows a higher values and strong hysteresis. On the other hand, slower scan speeds conditions the *PCE* decrease and smaller hysteresis is observed. Many reports on perovskite solar cells have demonstrated hysteresis at various scanning rates^7–9^. In general, the hysteresis is attributed to the slow dynamic processes of charge trapping and de-trapping and unbalanced electron and hole transport because of low quality of the perovskite films and defects at the interfaces, which act as capacitors. Then, formed capacitor plays an important role in the origin of the hysteresis. This is because a capacitor takes time to charge and discharge. In the absence of charge accumulation, the hysteresis is effectively suppressed as demonstrated by inverted structure devices using PCBM, because of better contacts with organic molecules. The interface TiO_2_/CH_3_NH_3_PbI_3_ is the main origin of the hysteresis in the PSCs due to the lattice mismatch between two layers, which affects the rate of the electrons transfer at the interface^25,26^. The *I-V* curves at different scan rates for the real devices (Fig. 2) supported our previous report describing two interfacial capacitances and conclude that the hysteresis originates from these interfacial capacitances.

Moreover, our results are supported by already published data the of devices with TiO_2_ layer, without perovskite layer inside and by CsPbI_3_ based solar cells which shows the hysteresis in the *I-V* curves^27,28^. For these two examples, the ion migration is not applicable and it cannot be the main origin of the hysteresis. Hysteretic phenomena have been also been observed in other types of solar cells including CIGS, CdTe, silicon and dye-sensitized solar cells that exhibit high internal capacitances. In these devices the behavior is related to the accumulation of charge carriers at the junction, or by the existence of the defect states^11,18,29^ rather than ion-migration. Interfacial contacts plays important role in the electrical proprieties of the devices. The interfaces are sites of defects in a periodic crystal due to breaking of the periodicity. This acts as carrier trap states and cause static charge accumulation, leading to increased recombination^30,31^. Defects at the interfaces generate the capacitance, which can be one possible cause of the hysteresis observed in the *I-V* curves for PSC.

### Maximum power point tracking (MPPT) for high capacitance based solar cells

*I-V* curve measurement is valid for most of conventional solar cells such as c-Si which have very fast response time compared to voltage scanning rate. In other words, *I-V* based characterizing technique is applicable to a cell that has *I-V* curves independent of scan speed and direction. But unfortunately, most of organic solar cells including PSC and DSC show slower response behavior and hysteresis in *I-V* curves, as we have observed in our experiments (Fig. 1). In such cases the *I-V* curve shape depends on the scan speed and direction and thus *P*_max_ also depends on the scan speed and direction conditions. Data points in the *I-V* curve represent the instantaneous voltage and corresponding current value of the solar cell. If we keep the cell at a particular output condition (fixed voltage) it cannot produce the same current as observed on scans. Therefore, the *P*_max_ value obtained from an *I-V* curve does not represent the true output value of the cell. In order to avoid the wrong evaluation of the *PCE* of the DSC and PSC we have constructed a machine for tracking the maximum power point, *P*_max_. The measurement essentially extracts the maximum power of each cell at different light intensities, which was monitored by the LED solar simulator. Among all the papers already proposed we used hill climbing, and perturb and observe methods (Figs S7 and S8).

**Figure 3:**
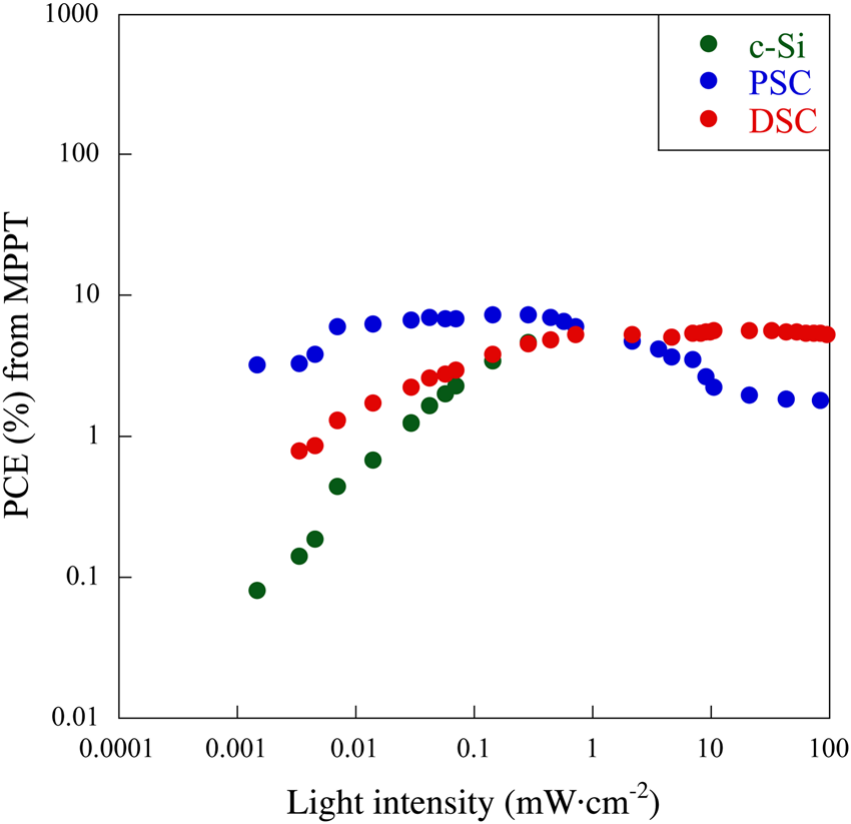
Power conversion efficiencies for c-Si solar cell (green curve), DSCs (red curve) and Perovskite solar cell (blue curve) at different light intensity extracted from MPPT.

Generally, as we have mentioned in the introduction, maximum power point tracking (MPPT) is used for maximizing power output of large solar arrays. It is implemented in the DC-DC converting stage of the solar inverters which handles several hundreds to thousands of watt output power and incorporate fast switching technique. Electronic circuits in the inverter uses several watts of solar generated power to operate. These industrial inverters with built in MPPT function needs more than 10 V and 1 A of output from the solar panel^32,33^. Furthermore, this technology cannot be used to measure exact output power of the solar cell since part of the power is utilize by the inverter itself. There are different well known algorithms to track MPP^34^. We are using modified hill climbing technique experimentally optimized for PSC cells with high capacitive behavior. The new MPPT analyzer we report here, maintain the cell at maximum power point (*P*_max_) by using externally powered electronics and can thus precisely measure the maximum output power of the cell for values as low as a few pW different as reported MPPT analyzer^34^. Unlike the MPPT tracking technique used in industrial solar power inverters, this analyzer is not using switching technique and it is purely DC bias (continuous), which makes it possible to maintain the cell at maximum power point. We believe the direct measurement of *P*_max_ solves the issue that originates from the over/underestimation of the *PCEs* for devices showing hysteresis in the *I-V* curves, and provides trustworthy unique evaluation results for high capacitive solar cells. This technique answers the issues with current evaluation technique by continuously keeping the cell at the *P*_max_ condition. The maximum output power (include the voltage and current at the *P*_max_ and energy conversion efficiency) of the solar cell is plotted with time. Therefore, the time and direction dependent *I-V* behavior of high capacitance cells is no longer an issue since we can track how the maximum output power (including voltage and current at the *P*_max_) changes with time.

c-Si solar cell has been used as reference for comparing the *P*_max_ tracking with PSC and DSC. The obtained data from MPPT measurements are shown in the Fig. 3.

In order to avoid the degradation of PSCs, *P*_max_ tracking was conducted from 10^−4^ to 10^2^ mW·cm^−2^. In the case of c-Si solar cell and DSC the measurement were conducted from 10^2^ to 10^−4^ mW·cm^−2^. The final *PCE* values were calculated from the MPPT recorded after stabilization of the devices (after 500 s).

We found that the *PCE* of c-Si solar cell decreases drastically with decreasing light intensity from 10^2^ to 10^−4^ mW·cm^−2^. A similar trend was observed for PSC and DSC but the order of the decrease is smaller than for the c-Si solar cell. Moreover, the photovoltaic response below 10^−2^ mW·cm^−2^ light intensity is low without erroneous value of *PCE* as we observed in the *I-V* curves measurement (Fig. 1). Due to the instability of the PSC under the strong light intensities the efficiencies decreases during the MPPT tracking from 1 to 10^2^ mW·cm^−2^. In the case of DSC a decrease in *PCE* below 1 mW·cm^−2^ and stabilized *PCE* from 1 to 10^2^ mW·cm^−2^ is observed. Hence a combination of both DSC and PSC can be a solution for industrial application for wider range of light intensities from 10^2^ to 10^−2^ mW·cm^−2^, PSC can be a good option for low light applications such as imaging sensor.

In the present work, we constructed a LED solar simulator and MPPT tracking machines for evaluation of the power conversion efficiency of perovskite and dye-sensitized solar cells under wider range of light intensity including indoor light illumination (from 10^2^ to 10^−4^ mW·cm^−2^). The linear relation of *J*_sc_ and light intensity for c-Si solar cell measured with a *I-V* machine confirms the reliability of the calibration of the light intensity. PSC and DSC are more stable solar cells at low light intensity as compared to c-Si solar cell and give a photovoltaic response until light intensities as low as 10^−2^ mW·cm^−2^. A constant current is observed for PSC and DSC when the light intensity is decreased to lower than 10^−2^ mW·cm^−2^. This is assigned to originate from a capacitive current that overestimates the *PCE* of more that 100% for PSC and over 10% for DSC. The constant open circuit voltage and short circuit current in the reverse scan condition for PSC and DSC at low light intensity originates from internal capacitance of the devices, which gives small amount of current, called capacitive current, due to the charging-discharging of the capacitance under applied bias. We found the capacitance equal to 4.66 μF·cm^−2^ for DSC, which is one order of magnitude lower that the capacitance of PSC, 37.9 μF·cm^−2^. This difference likely related to the interfacial contacts and results in the hysteresis gap observed in *I-V* measurements for both devices. In the case of c-Si solar cells no hysteresis is observed and the capacitance is equal to 0 μF·cm^−2^. Our experiment shows direct evidence of the effect of the capacitance during the *I-V* measurement and the origin of the hysteresis in organic solar cells including PSC and DSC. PSC can be categorized as high capacitance solar cells. In order to avoid the overestimation of PSC and DSC, we measure the MPTT. This approach gives more accurately *PCEs* of those solar cells, which clears the ambiguity about *I-V* evaluation of high capacitive solar cells showing hysteresis during *I-V* measurement. Compared to c-Si solar cell, PSCs exhibit promising stable photoresponse in the range from 1 to 10^−2^ mW·cm^−2^, with a slight decrease in the range from 10^−2^ to 10^−3^ mW·cm^−2^ and are therefore of great interest from a practical application point of view. No photoresponce was registered for any of the three types of solar cells bellow light intensities 10^−3^ mW·cm^−2^.

We believe that LED solar simulators with wider range of light intensities, combined with the MPPT tracking technique will be acknowledge as standard evaluation techniques capacitive organic photovoltaics showing hysteresis behavior during *I-V* measurements.

## Methods

### Device preparation

In this study, standard c-Si solar cells, VLSI Standards, Inc. SRC-1000-RTD-XL-J calibrated by TÜV Rheinland under IEC 60904-2 testing condition was used. Our perovskite solar cell has planar with the TiO_2_/CH_3_NH_3_PbI_3_/spiro-OMeTAD/Au structure^35^. Sealed liquid-state dye sensitized solar cell with the configuration FTO/TiO_2_ (meso)/Dye (N719)/3-MPN electrolyte/Pt/ITO were used for our experiment.

### Device evaluation

Solar simulator using LED lamps as low cost and easily tunable light source (to the intensity 10^−3^ mW·cm^−2^) has been specially designed and constructed for this work. 31 kinds with 54 LED bulbs with different wavelengths were arrayed along with a dome shaped framework to focus on the stage (Supplementary Fig. 9a). The collimator lens was equipped for each LED to keep high intensity and parallelism. The resulting spectra match was in the range 0.95–1.05 from 400–900 nm at 10^2^ mW·cm^−2^, followed by the IEC 60904-9 Edition 2 and ASTM E927-10 standards for common specifications of solar simulators as shown in the Supplementary Fig. 9(b) and Supplementary Table 1. Non-uniformity of irradiance was maintained ±5% at 100 mm square and reached less than ±2% at 30 mm square (Supplementary Fig. 9c). Temporal instability of the light intensity was kept less than ±1%. In order to stabilize the change of properties of the lamp with time, an air-cooling unit was attached for each LED bulbs.

Moreover, non-standard testing conditions at low light intensity were carefully tuned by monitoring spectroradiometer (S-2440 model II, SOMA). The resulting spectra mismatch was in the range less than 10% from 400–900 nm at 10^−3^ mW·cm^−2^ illumination, as shown in Table S1. The signal noise in the dark conditions was below 1.2 × 10^−6^ mW·cm^−2^·nm^−1^ in the range of 300–1100 nm and covered our lowest irradiation condition 1.0 × 10^−4^ mW·cm^−2^. Light intensity below 10^−3^ mW·cm^−2^ has been monitored by using the stainless mesh and intensity decreased to 10^−4^ mW·cm^−2^. For accurately evaluation of solar cells the PV Power Analyzer (VK-PA-Pico, SPD laboratory) is proposed for tracking maximum power point under varying light intensities monitored by the LED solar simulator.

Active area of c-Si solar cell, PSC and DSC, for *I-V* measurement were fixed at 1 cm^2^, 0.0314 cm^2^, 0.25 cm^2^, respectively. The *J-V* (from −0.05 to 1.2 V) and reverse scan (from 1.2 to −0.05 V) directions simultaneously. The scan step value and scan rate are 0.04 V and 100 mV·s^−1^, respectively.

Maximum power point tracking (MPPT) technique explained in this paper used source measure unit (in constant voltage model).

To avoid the voltage drop across the current measuring circuitry, high precision op-amp (input offset 0.5 μV) based feedback ammeter was incorporated in the instrument. In order to avoid leakage current through the voltage measuring unit, extremely high input impedance Op-amp buffer was used in voltage measuring section (Fig. S7). Op-amp used for pico-ammeter stage (as feedback ammeter) is LTC1050AC from “Linear Technology”. The data sheet shows ±20 pA input offset current and ±0.5 μ V input offset voltage. Op-amp output error voltage due to those two factors were compensated by the firmware when we perform external calibration in our MPPT-Pico. Voltage reference chip is AD780BN from “Analog Devices”. Analog to digital converter (16 bit, non linearity 0.003%) AD7705 from “Analog Devices”. According to minimum measured current value 132 nA at light intensity 2.51 × 10^−4^ mW, measuring range should be 60 nA with theoretical resolution 2 pA. In the worst case maximum error would be 0.05% (about 30 pA) for this measuring range. Typical error should be 0.01% from full scale value of the range. Therefore 132 nA reading not showing any uncertain digits.

## Electronic supplementary material

Supplementary Information
