# Supplementary material for: Determination of unique power conversion efficiency of solar cell showing hysteresis in the I-V curve under various light intensities
Source: Sci Rep. 2017 Sep 18;7:11790. doi: 10.1038/s41598-017-10953-3 (PMC5603576; doi:10.1038/s41598-017-10953-3)
Supplement: Supplementary file 1 — Supplementary Information [file 41598_2017_10953_MOESM1_ESM.docx]

**Supplementary information**

**Determination of unique power conversion efficiency of solar cell showing hysteresis in the I-V curve under various light intensities**

Ludmila Cojocaru^1^*, Satoshi Uchida^2^*, Koichi Tamaki^1^, Piyankarage V. V. Jayaweera^3^, Shoji Kaneko^3^, Jotaro Nakazaki^1^, Takaya Kubo^1^, Hiroshi Segawa^1,4^*

^1^Research Center for Advanced Science and Technology, The University of Tokyo, Komaba 4-6-1, Meguro-ku, Tokyo 153-8904, Japan

^2^Komaba Organization for Educational Excellence, Faculty of Arts and Sciences, The University of Tokyo, Komaba 3-8-1, Meguro-ku, Tokyo 153-8902, Japan

^3^SPD Laboratory, Inc., Johoku 2-35-1, Naka-ku, Hamamatsu 432-8011, Japan

^4^Department of General Systems Studies, Graduate School of Arts and Sciences, The University of Tokyo, Komaba 3-8-1, Meguro-ku, Tokyo 153-8902, Japan

* Corresponding author e-mail: [uchida@rcast.u-tokyo.ac.jp](mailto:uchida@rcast.u-tokyo.ac.jp), [cojocaru@dsc.rcast.u-tokyo.ac.jp](mailto:cojocaru@dsc.rcast.u-tokyo.ac.jp), [csegawa@mail.ecc.u-tokyo.ac.jp](mailto:csegawa@mail.ecc.u-tokyo.ac.jp)

a) DSC
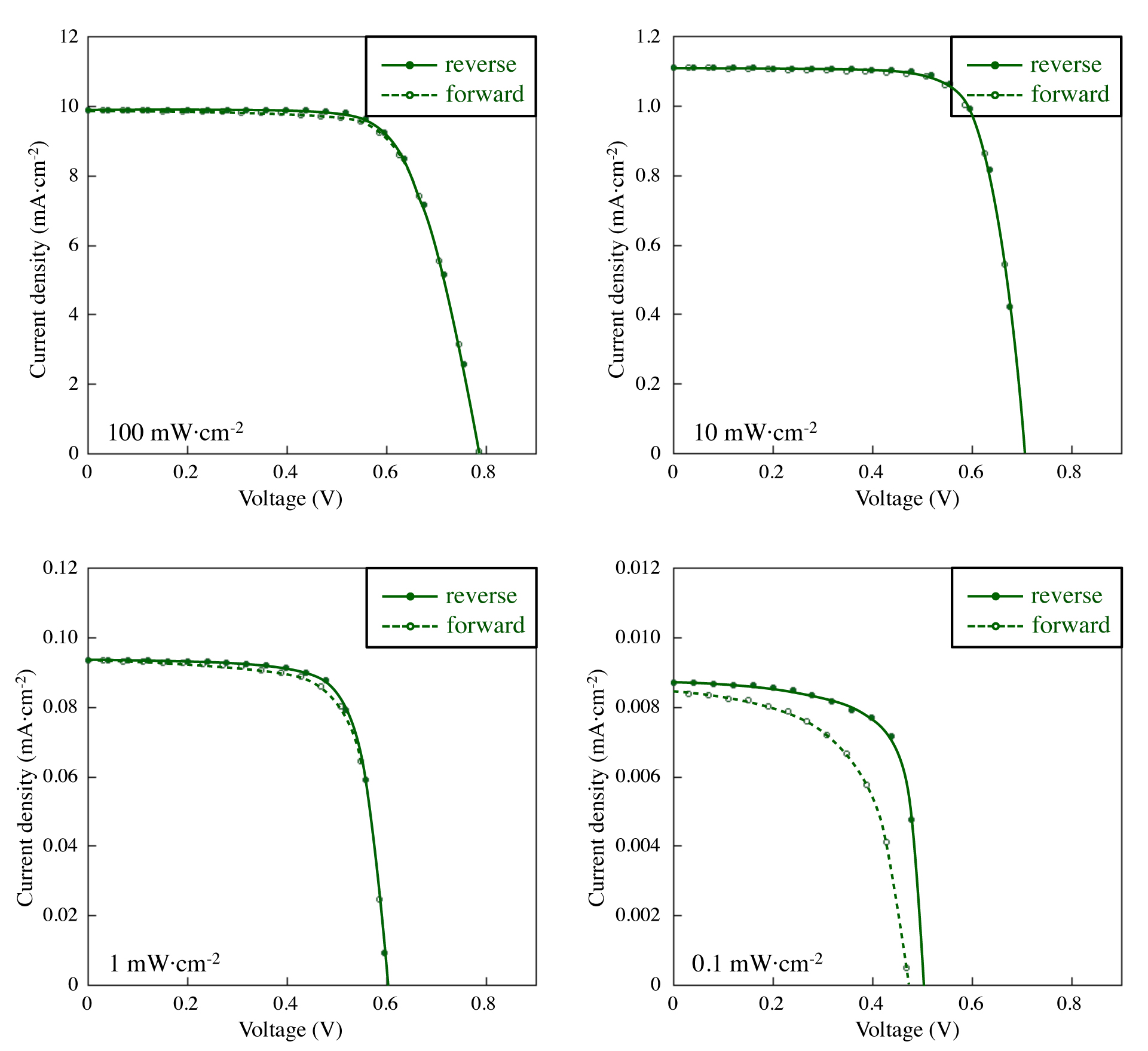


(b) PSC


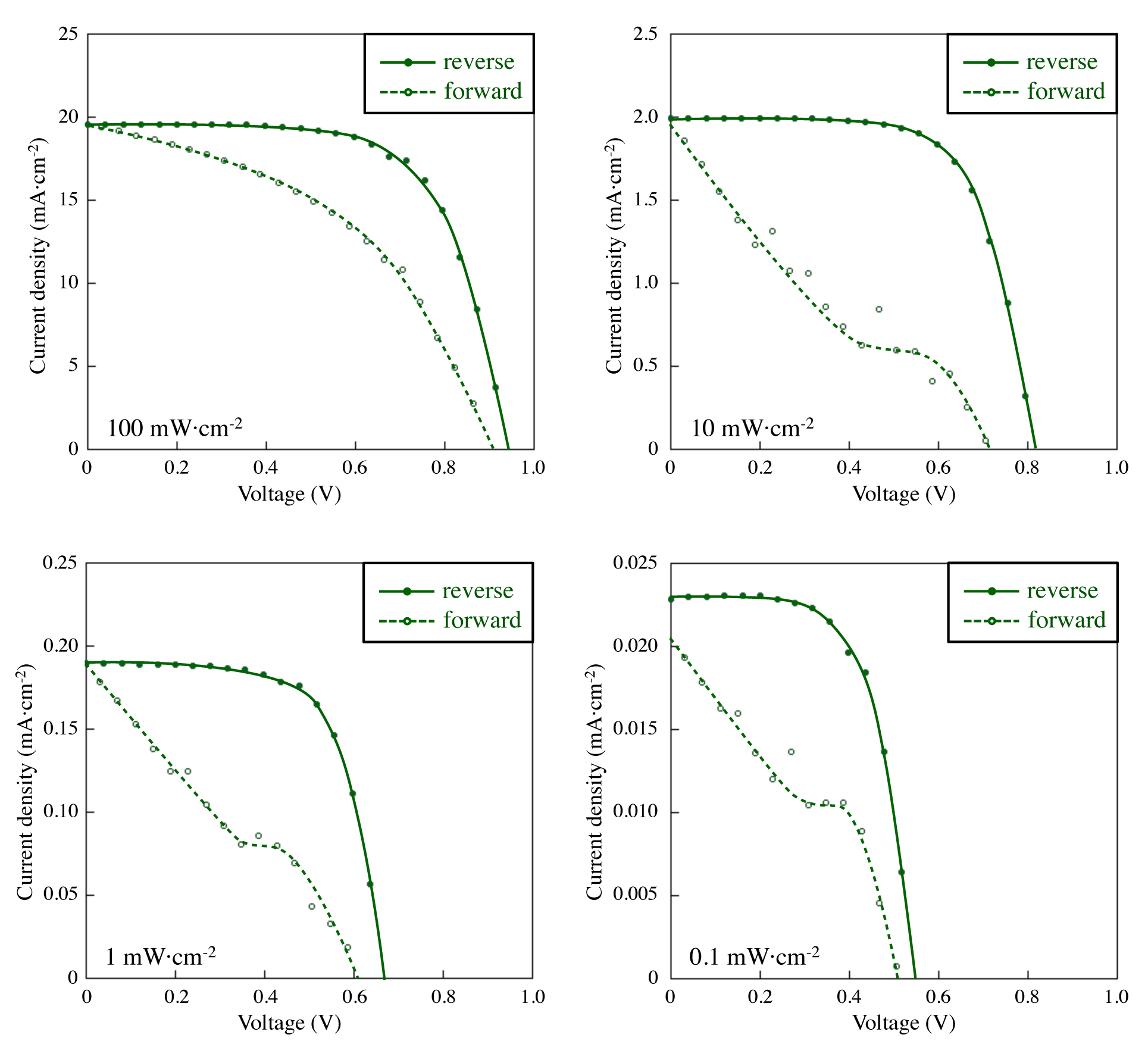


Supplementary Figure S1. I-V curves for DSC (a) and PSC (b) at different light intensity conditions. For DSC the I-V measurement was performed from 10^2^mW·cm^-2^ to 10^-4^ mW·cm^-2^, for PSC from 10^-4^ mW·cm^-2^ to 10^2^mW·cm^-2^ (scan rate 100mV·s^-1^).


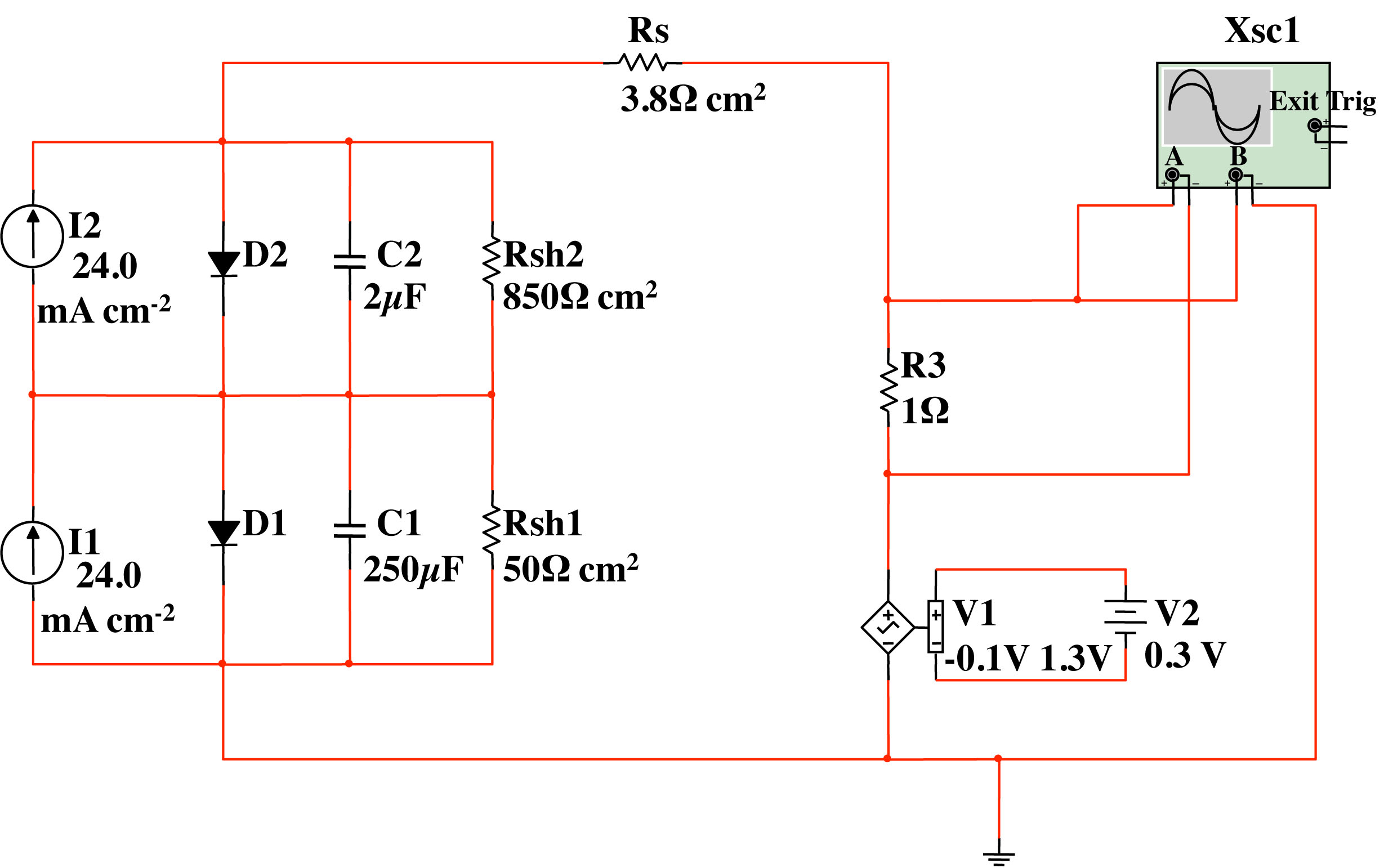


**Supplementary Figure S2.** Equivalent circuit model of perovskite solar cell with double diodes, capacitances, Rsh and single Rs has been used for simulation of the I-V curves with strong hysteresis.

***During reverse I-V scan (fig. S3 a):***

**I-V curve in the IV^th^ quadrant: (***V_set_>V_oc_*)

Current flow in to the cell and internal capacitor discharging, total output current can be expressed with the formula 1.

**I-V curve in the I^st^ quadrant:** *(0<V_set_<V_oc_)*

Current flow out from the cell and internal capacitor discharging, total output current can be expressed by the formula 2.

**I-V curve in the II^nd^ quadrant:** *(V_set_<0)*

Current flow out from the cell and internal capacitors charging to opposite direction, total output current can be expressed with formula 3.

*I_input_ = I_cell-bias_ - I_cap.discharge_*  (1)

*I_output_ = I_photo_ + I_cap.discharge_*  (2)

*I_output_ = I_photo_ + I_cap.charge_*  (3)

***During forward I-V scan (fig. S3 b):***

**I-V curve in the II^nd^ quadrant:** *(V_set_<0)*

Current flow out from the cell and internal capacitors discharging, total output current can be expressed with formula 4.

**I-V curve in the I^st^ quadrant:** *(0<V_set_<V_oc_)*

Current flow out from the cell and internal capacitor charging, total output current can be expressed by the formula 5.

**I-V curve in the IV^th^ quadrant: (***V_set_>V_oc_*)

Current flow in to the cell and internal capacitor charging, total output current can be expressed with the formula 6.

*I_output_ = I_photo_ - I_cap.discharge_* (4)

*I_output_ = I_photo_ - I_cap.charge_* (5)

*I_input_ = I_cell-bias_ + I_cap.charge_*  (6)

(a)


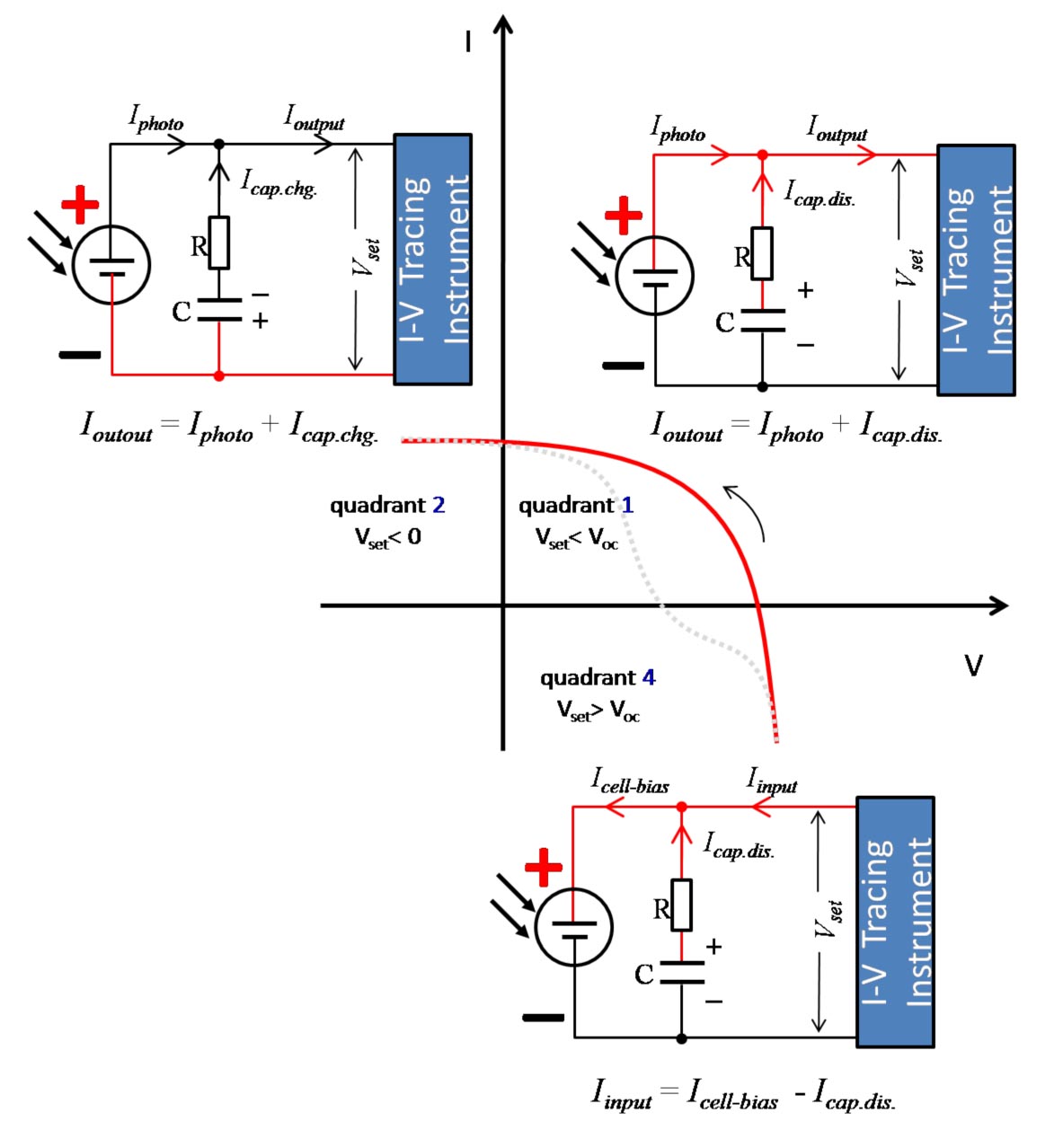


(b)


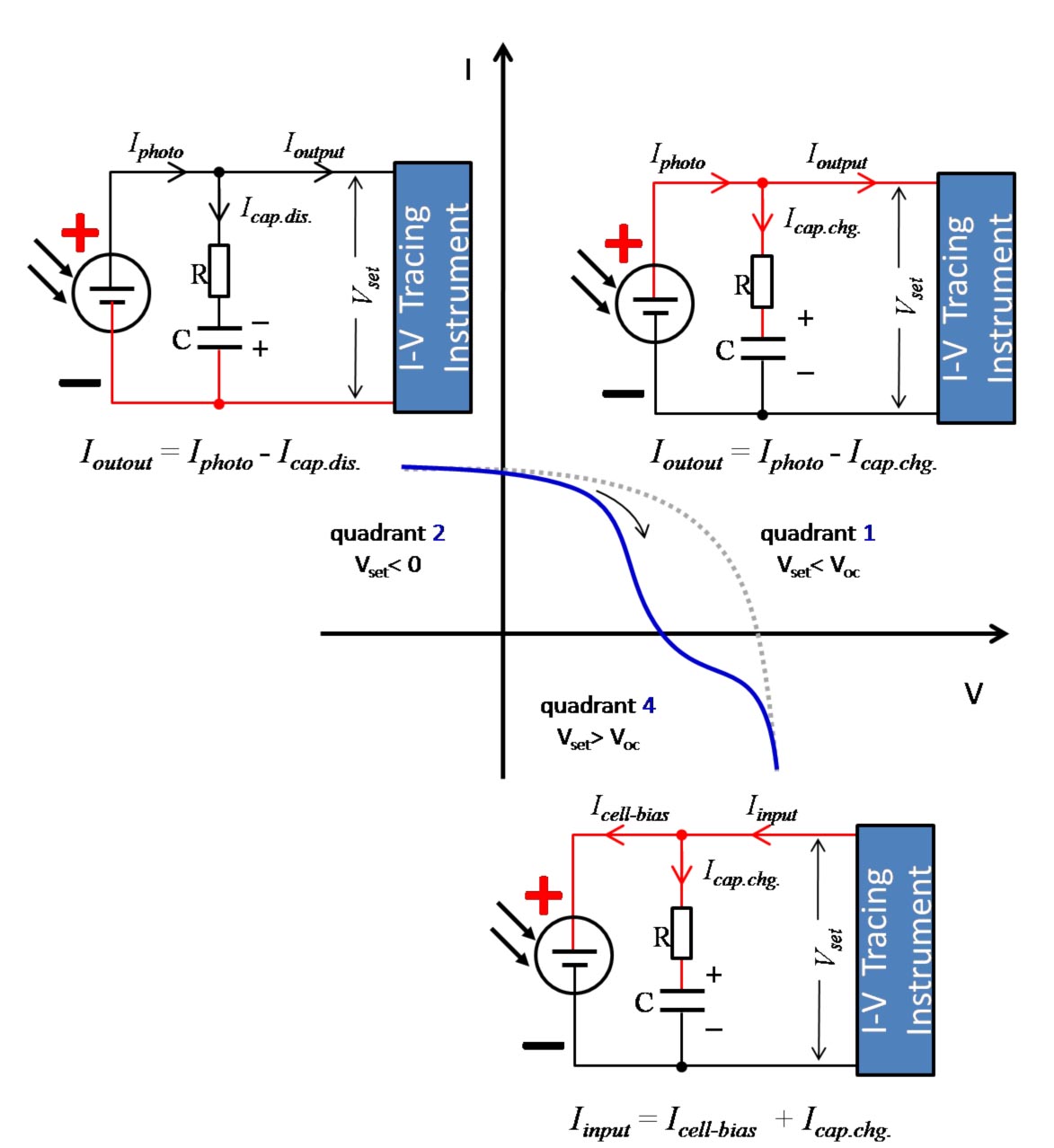


**Supplementary Figure S3.** Capacitance and current flow for (a) reverse and (b) forward scan conditions during I-V measurement for solar cells.


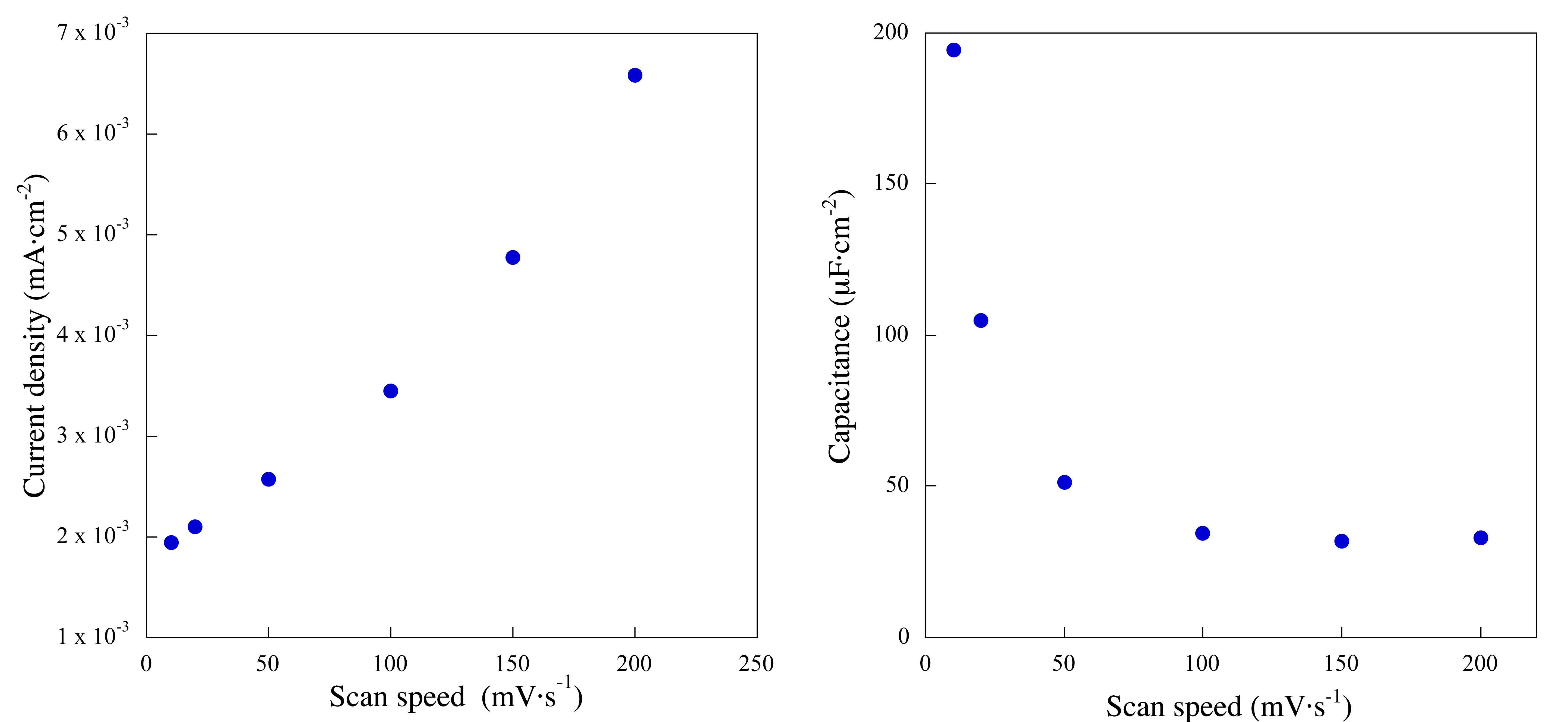


**Supplementary Figure S4.** Plotted of capacitive current and capacitance versus scan rate for perovskite solar cell at light intensity of 6.98×10^-3^ mW·cm^-2^.

(a) (b)


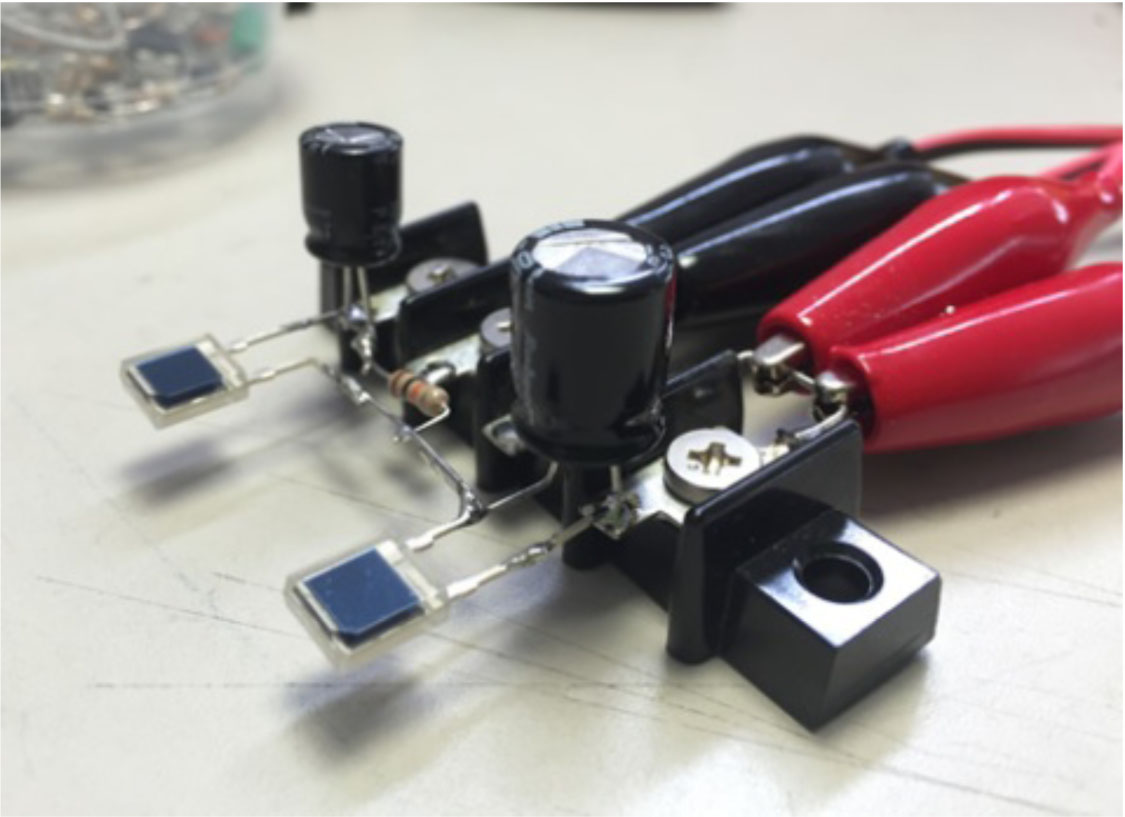

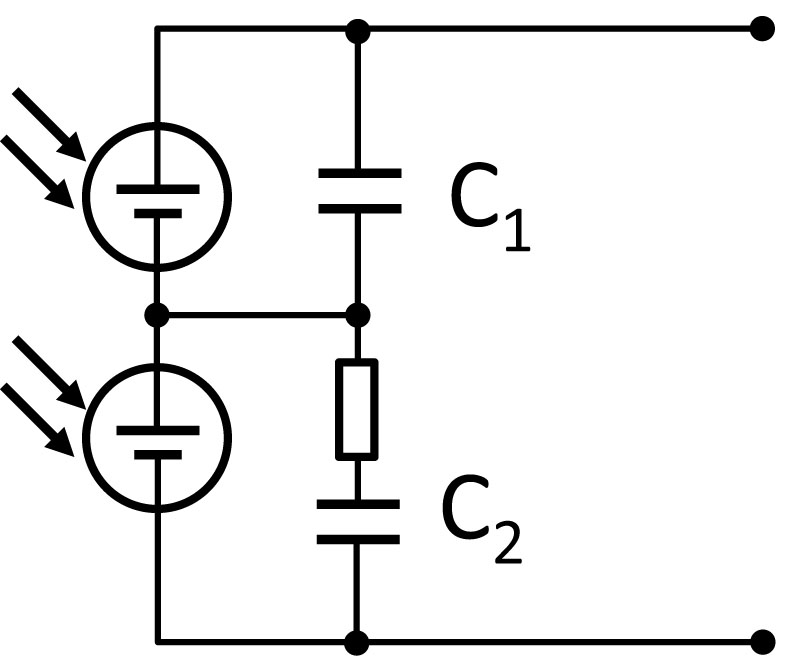


**Supplementary Figure S5.** (a) Real physical device constructed based on (b) equivalent circuit with double diodes based on silicon solar cell, two different capacitances and resistance.


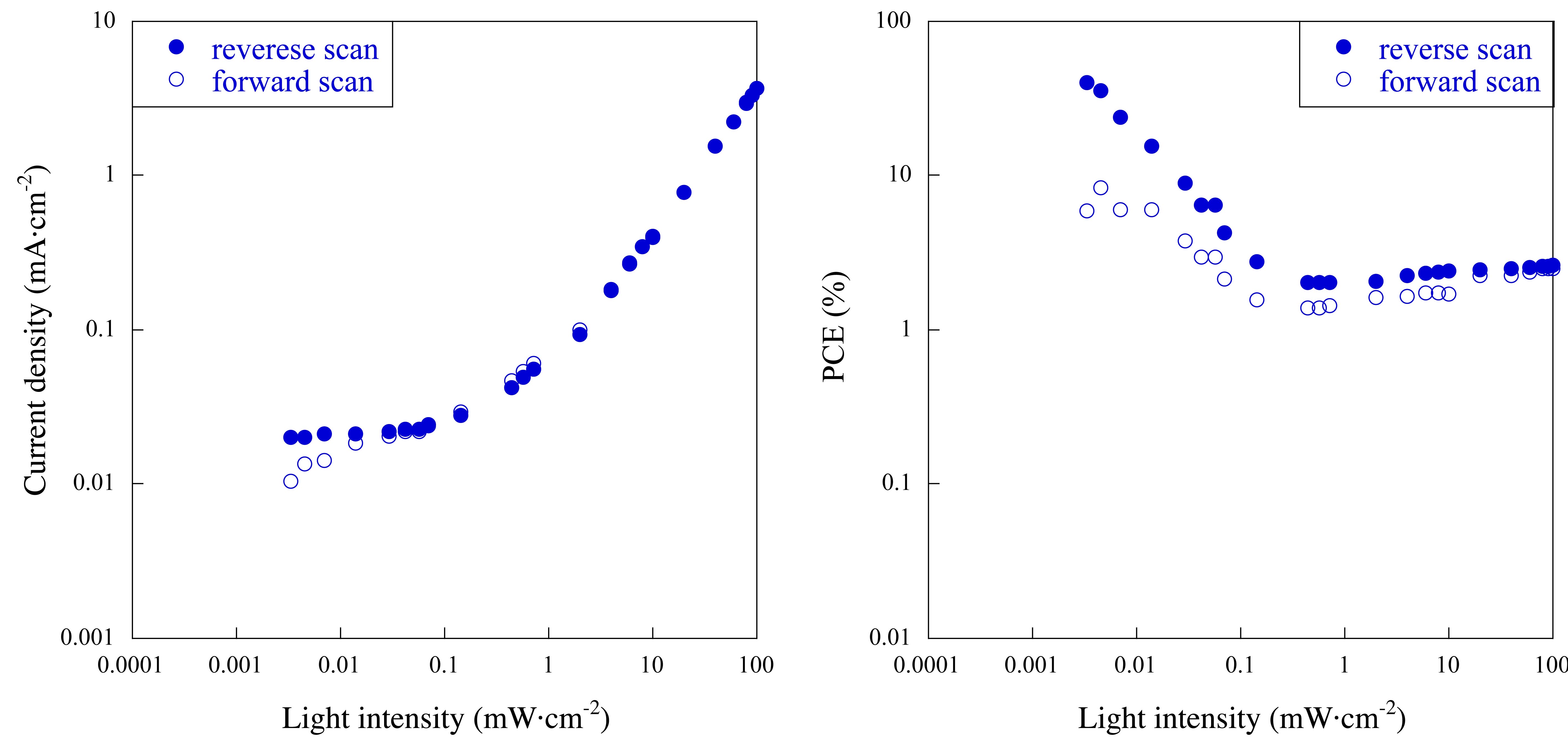


**Supplementary Figure S6.** Evaluation of the J_sc_ and PCE of the real device constructed based on double capacitor equivalent circuit at different light intensity, closed circles: reverse scan and open circles: forward scan conditions.

**Maximum Power Point Tracking Method (MPPT).**


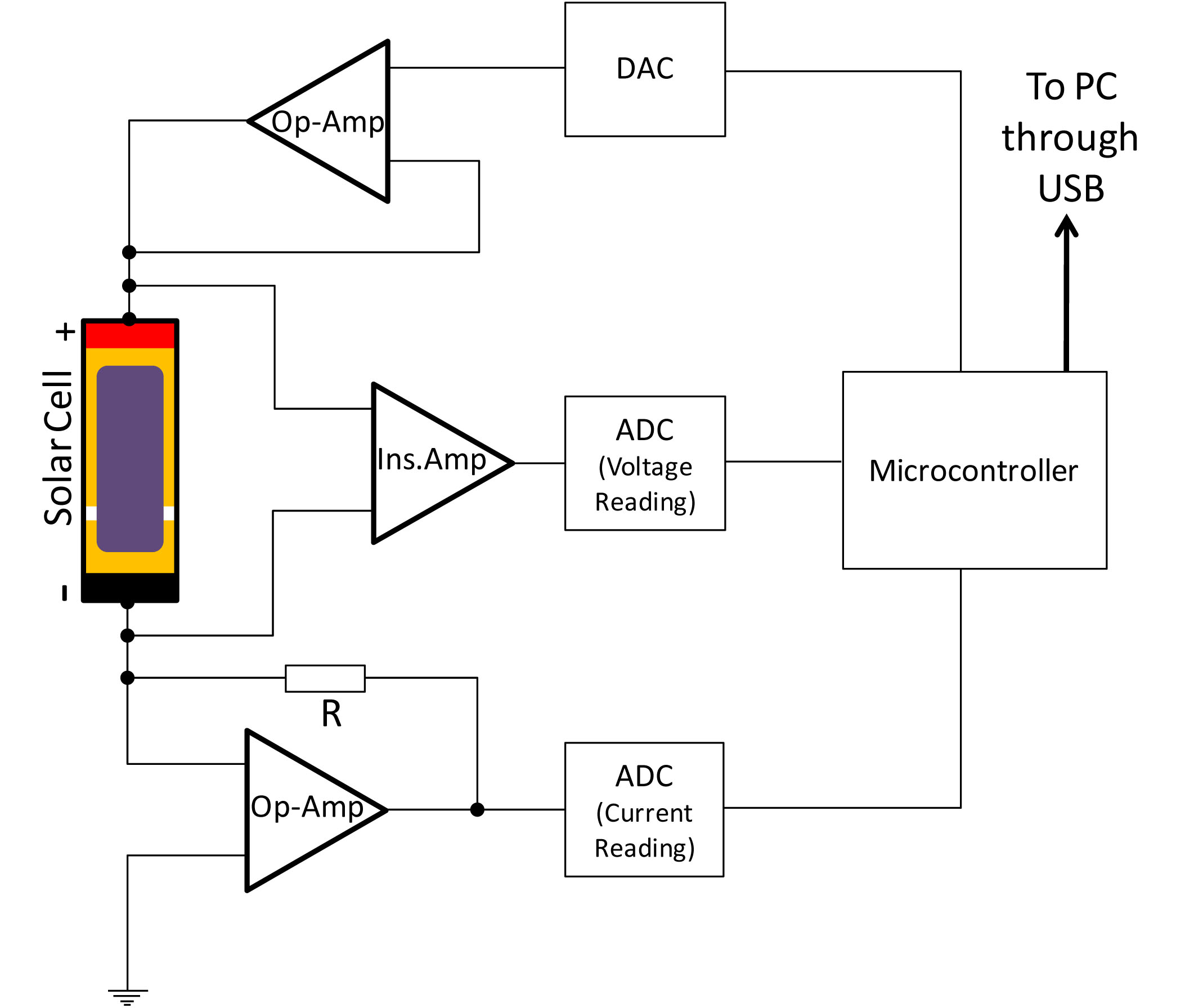


**Supplementary Figure S7.** Simplified block diagram of the MPPT solar cell characterization system. Basic components are operational amplifiers (Op-Amp), instrumentation amplifier (Ins. Amp), analog to digital converters (ADC), digital to analog converter (DAC), and microcontroller with MPPT firmware program.


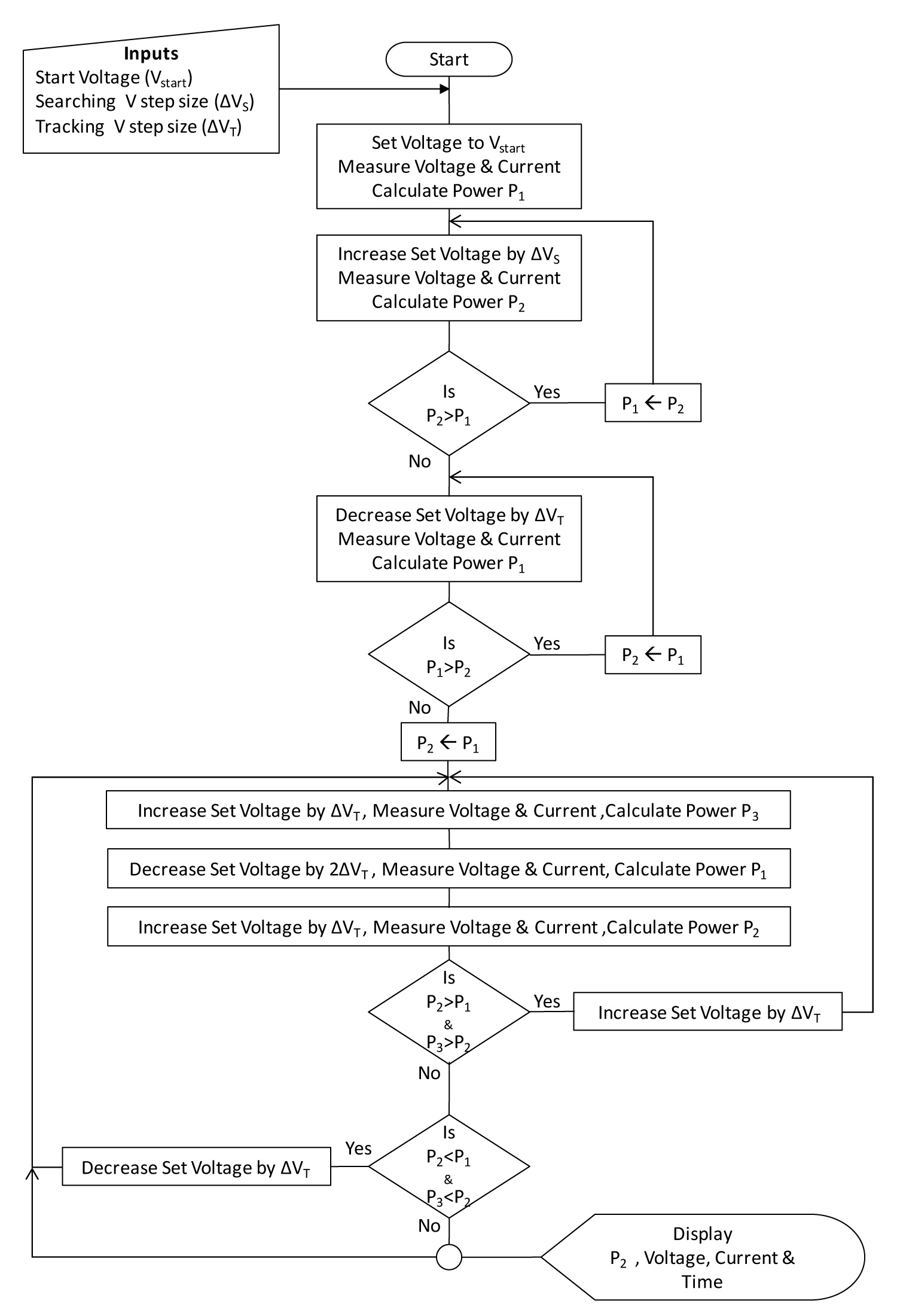


**Supplementary Figure S8.** Flow chart of the MPPT algorithm.

(a)


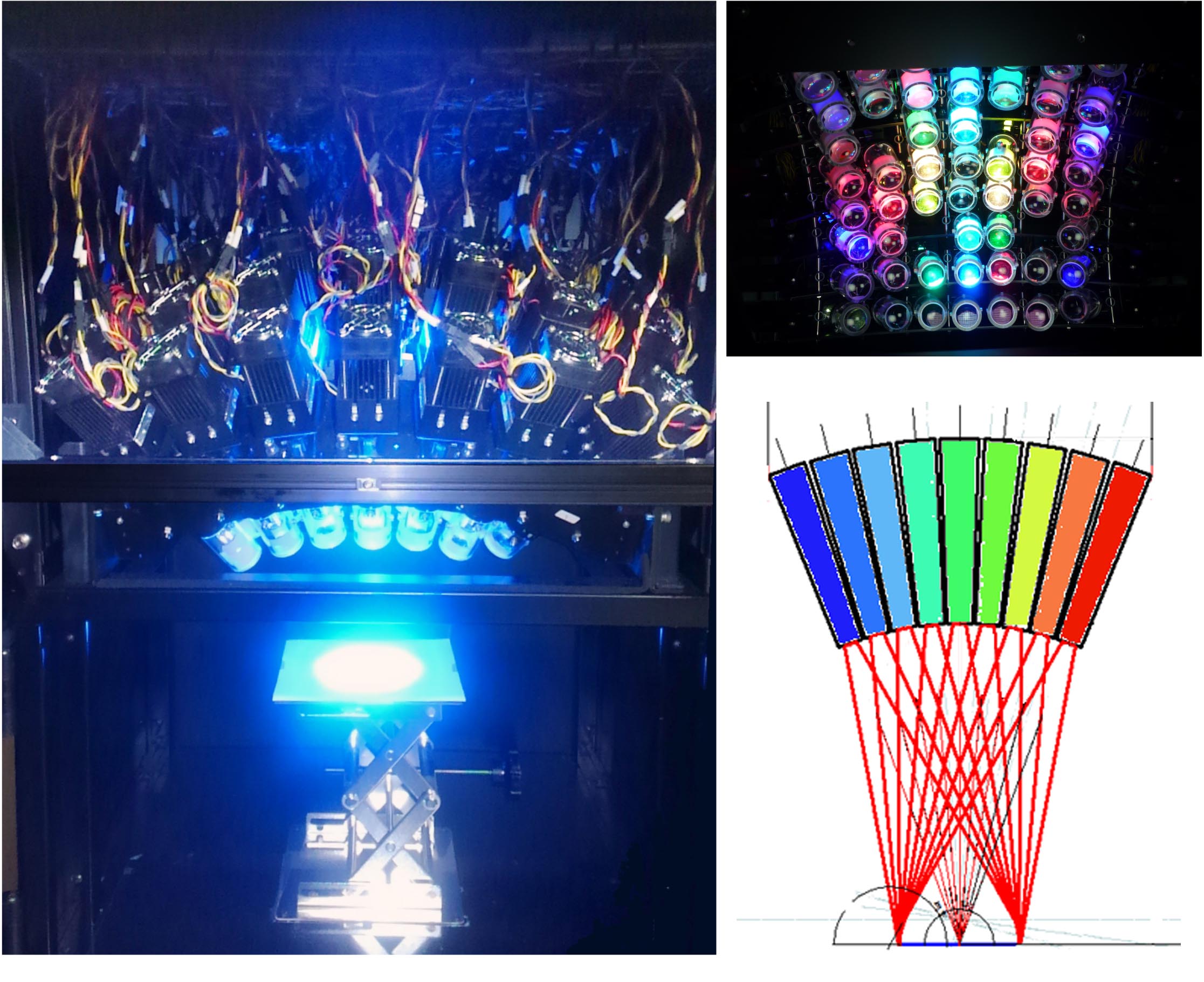


(b)


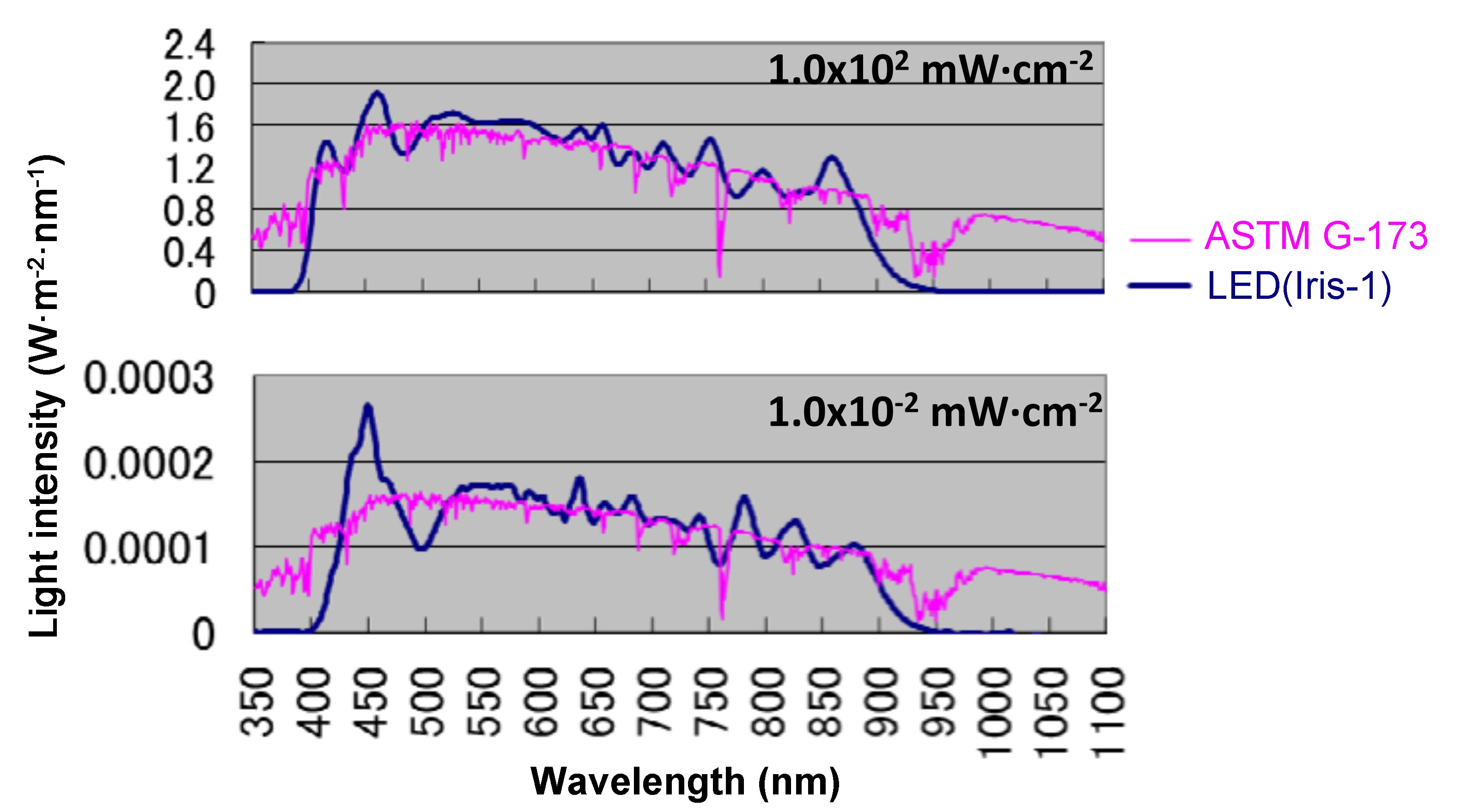


**Table S.1** LED solar simulator adjustment parameters at 10^-2^ mW·cm^-2^.

| Wavelength interval,  nm | Full solar spectrum, W/m^2^/nm | % | IRIS solar spectrum, W/m^2^/nm | % | Spectral match | % |
| --- | --- | --- | --- | --- | --- | --- |
| 400-450 | 0.0063 | 9.70 | 0.0064 | 9.48 | 0.98 | -2.3 |
| 450-500 | 0.0079 | 12.2 | 0.0079 | 11.8 | 0.97 | -3.3 |
| 500-550 | 0.0078 | 11.9 | 0.0076 | 11.4 | 0.95 | -5.0 |
| 550-600 | 0.0076 | 11.7 | 0.0084 | 12.5 | 1.07 | 7.4 |
| 600-650 | 0.0073 | 11.3 | 0.0076 | 11.2 | 1.00 | -0.3 |
| 650-700 | 0.0068 | 10.5 | 0.0073 | 10.8 | 1.03 | 2.9 |
| 700-750 | 0.0061 | 9.42 | 0.0065 | 9.64 | 1.02 | 2.3 |
| 750-800 | 0.0054 | 8.34 | 0.0057 | 8.51 | 1.02 | 2.1 |
| 800-850 | 0.0050 | 7.64 | 0.0053 | 7.93 | 1.04 | 3.8 |
| 850-900 | 0.0046 | 7.16 | 0.0044 | 6.57 | 0.92 | -8.3 |

(c)


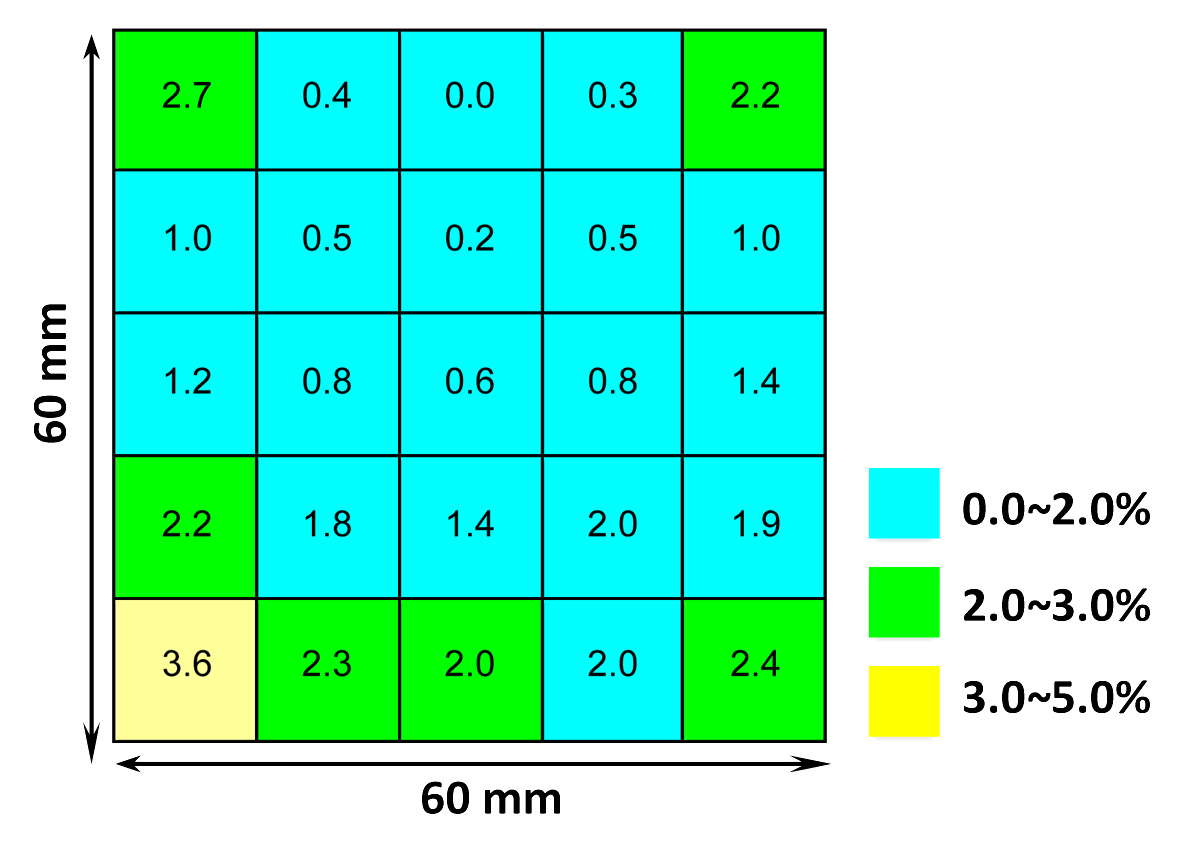


Supplementary Figure S9. (a) LED light orientation and distribution, (b) light intensity distribution for LED and ASTM G-173 (c) uniformity and relative intensity deviation on the 60x60 mm area.
